# Can Green-Technology Innovation Reduce Atmospheric Environmental Pollution?

**DOI:** 10.3390/toxics11050403

**Published:** 2023-04-24

**Authors:** Jingkun Zhou, Yunkai Zhou, Xu Bai

**Affiliations:** 1 School of Business, Ludong University, 186 Hongqizhong Road, Yantai 264025, China; 2 Adam Smith Business School, University of Glasgow, Glasgow G12 8QQ, UK; 3 School of Public Administration, Hebei University of Economics and Business, Shijiazhuang 050061, China; baixu092069@163.com

**Keywords:** green-technology innovation, atmospheric environmental pollution, spatial spillover effect, threshold characteristic

## Abstract

Rapid economic growth leads to such problems as resource scarcity and environmental degradation. Local governments successively take measures such as technological innovation to solve atmospheric environmental pollution; however, technological innovation fails to fundamentally alleviate atmospheric environmental pollution. Therefore, local governments come to realize the importance of green-technology innovation, which means an inevitable choice for various countries in the world to seek long-term development and win competitive advantage. Under such circumstances, this paper chooses the panel data of 30 provinces and regions in China from 2005 to 2018, takes environmental regulation as the threshold variable, and empirically analyzes the relationship between green-technology innovation and atmospheric environmental pollution by constructing a Spatial Measurement Model and Panel Regression Model. As evinced, green-technology innovation has a significant inhibitory effect and a spatial spillover effect on atmospheric environmental pollution. When environmental regulation reaches a level of intensity, green-technology innovation can effectively curb atmospheric environmental pollution. Accordingly, relevant parties should strengthen green-technology innovation, coordinate the development of the governance system of green-technology innovation, establish a joint prevention and control mechanism, increase the investment in green technology research and development, and augment the role of green-technology innovation.

## 1. Introduction

According to data from Bulletin of China’s Ecological Environment in 2021 released by the Ministry of Ecology and Environment, among 337 prefecture-level and above cities in China, 121 cities suffer excessive pollution in terms of ambient air quality, accounting for 35.7%, down by 3.5% over 2020 [1]. Nevertheless, the problem of atmospheric environmental pollution needs to be deeply investigated. Therefore, reducing atmospheric environmental pollution with green-technology innovations [2] and then promoting the high-quality development of China’s green economy signal the top priorities for future development. Simultaneously, can green-technology innovation effectively mitigate atmospheric environmental pollution? Does green-technology innovation form a spatial spillover effect with atmospheric environmental pollution? For one region, will green-technology innovation affect the environment of neighboring regions? This paper will examine or solve these questions.

### 1.1. Green-Technology

Since Brawn and Wield first introduced the concept of “green technology” in 1994 [3], the importance of innovation in this field has been increasingly recognized in response to the growing severity of environmental problems [4,5]. Green technology refers to the application of innovative and sustainable solutions to address environmental challenges and promote sustainable development. It encompasses a wide range of fields, including renewable energy, green building materials, energy-efficient transportation, and waste reduction.

In 2019, China’s National Development and Reform Commission and Ministry of Science and Technology issued the Guiding Opinions on Building a Market-oriented Green Technology Innovation System, which defined green technology innovation as an emerging technological innovation that reduces consumption, minimizes pollution, improves ecology, promotes the construction of an ecological civilization, and realizes harmonious coexistence between human beings and nature. This encompasses fields such as energy conservation and environmental protection, clean production, clean energy, ecological protection and restoration, urban and rural green infrastructure, and ecological agriculture, as well as technology in product design, production, consumption, and recycling. (https://www.ndrc.gov.cn/xxgk/zcfb/tz/201904/t20190419_962441.html (accessed on 10 March 2023)).

Currently, numerous nations across the globe are utilizing green technology to tackle environmental obstacles and advance sustainable development. For instance, many countries are embracing renewable energy sources such as solar and wind power to decrease dependence on fossil fuels and contain greenhouse gas emissions. Additionally, certain cities worldwide are employing eco-friendly building materials and designs to curtail energy consumption and foster a salubrious living environment. Green technology boasts several benefits. Firstly, it can notably diminish carbon emissions, enhance air and water quality, and assuage climate change. Secondly, green technology can stimulate sustainable development and buttress economic growth. Thirdly, it can assist countries and communities to curtail their reliance on fossil fuels, thereby economizing costs and heightening energy security. Nevertheless, green technology also encompasses some drawbacks. Firstly, elevated initial investment costs may pose a significant hindrance to its widespread adoption. Secondly, green technology typically necessitates significant technological advancements, which can be daunting. Finally, certain green technologies (such as biofuels) may yield inadvertent negative impacts on the environment and society. In conclusion, green technology holds the potential to combat diverse environmental challenges and advance sustainable development. Nonetheless, its benefits and drawbacks must be astutely evaluated, and its implementation must be ensured to be sustainable and salutary in the long run.

### 1.2. The Relationship between Green-Technology Innovation and Atmospheric Environmental Pollution

**Figure 1:**
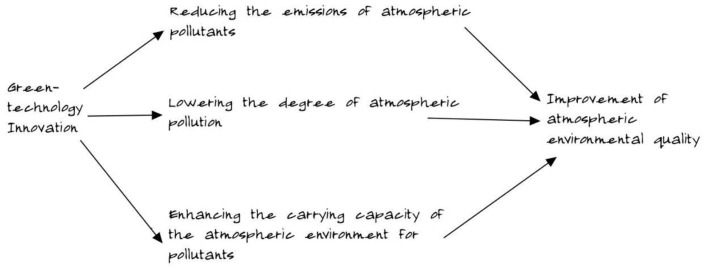
Schematic diagram illustrating the relationship between green technology innovation and air pollution.

As illustrated in Figure 1, the relationship between green technology innovation and air pollution can be described as follows: green technology innovation can improve the quality of the atmosphere by reducing the number of air pollutants emitted, decreasing the level of air pollution, and increasing the carrying capacity of the atmosphere. However, the relationship between green technology innovation and air pollution has caused controversy in the academic community, with scholars holding differing opinions on the matter. One view is that green technology innovation can improve the state of atmospheric pollution. Carrion-Flores and Innes (2010) assert that green technology innovation can improve environmental outcomes and to some extent help curb atmospheric pollution [6]. Oyebanji and Kirikkaleli (2023) found that the adoption of green technology and green electricity significantly reduces environmental pollution, including atmospheric pollution. Specifically, they discovered that in countries with high levels of green technology and green electricity, levels of air pollutants such as nitrogen oxides (NO_x_) and sulfur dioxide (SO_2_) are lower [7]. Zhou Jieqi (2014) observed that green-technology innovation has a significant impact on the reduction of carbon emissions [8]. Nie Puyan (2015) researched the heterogeneity of the impact of market concentration and technological innovation on the intensity of industrial carbon emission, revealing that technological innovation can facilitate energy progress and reduce environmental pollution [9]. Lu Xuehuan et al. (2020) concluded that green-technology innovation effectively curbs fog-haze pollution [10]. The other view is that green-technology innovation cannot help to reduce atmospheric environmental pollution. Li Fen et al. (2017) suggested that technological innovation aggravates environmental pollution. Technological innovation improves production efficiency; however, it consumes a lot of resources and causes environmental pollution [11]. Chen Yang et al. (2019) disclosed that in some cities in the northeast and northwest regions, the level of technological innovation serves industrial development and fails to reduce environmental pollution [12]. Schmidt (2019) argued that the total amount of environmental pollution generally increases, and technological innovation will not necessarily decrease environmental pollution [13].

### 1.3. The Spatial Spillover Effect of Green-Technology Innovation on Atmospheric Environmental Pollution

The spatial spillover of atmospheric environmental pollution signifies that environmental pollution affects one region as well as the atmospheric environment of its neighboring regions. Green-technology innovation can inhibit atmospheric environment pollution in one region and its neighboring regions. In this regard, scholars have carried out relevant research. Liu Xiaohong (2019) examined the effect of green innovation on fog-haze pollution under a spatial correlation, demonstrating that the direct effect, indirect effect, and total effect of green innovation prove significantly negative. This means the rise of the green-innovation level can effectively curb fog-haze pollution in local and neighboring regions, with an obvious spatial spillover effect [14]. Lu Na et al. (2019) constructed a dynamic Spatial Durbin Model to empirically test the spatial spillover effect of groundbreaking low-carbon technological innovation on carbon emission, upholding that groundbreaking low-carbon technological innovation has a significant inhibitory effect on carbon emission and insignificant effect on carbon emission in neighboring regions [15]. Ren Yayun et al. (2020) believe that urban innovation in China can curb fog-haze, with a significant spatial spillover effect [16]. According to Wang et al. (2022), the increase in intergovernmental competition and fiscal decentralization may lead to more green technology innovation, which in turn reduces air pollution in innovative regions and their surrounding areas [17]. Lu Xuehuan et al. (2020) noticed that green-technology innovation can effectively reduce fog-haze pollution, with a positive spatial spillover effect [10].

### 1.4. The Threshold Effect of Green-Technology Innovation on Atmospheric Environmental Pollution

Presently, scholars have scrutinized the threshold effect of green-technology innovation on atmospheric environmental pollution. Specifically, this paper mainly takes the intensity of environmental regulation as the threshold variable and discusses the threshold effect of green-technology innovation on atmospheric environmental pollution. Lan and Munro (2014) showed that as the intensity of environmental regulation increases, the market demand for environmental-protection technology expands. Therefore, enterprises will invest more human and material resources in the research and development of environmental-protection technology to improve environmental quality [18]. Huang Tianhang et al. (2020) confirmed that technological innovation under environmental regulation plays a positive role in reducing environmental pollution [19]. Lu Xuehuan et al. (2020) noted that when the level of regional environmental regulation proves relatively high, green-technology innovation has a significant inhibitory effect on fog-haze pollution [10]. Wu Lichao et al. (2020) asserted that as the level of environmental regulation rises, green-technology innovation produces a more significant effect on improving environmental quality [20].

Currently, scholars highlight the research on the relationship between technological innovation and atmospheric environmental pollution yet ignore the research on green-technology innovation. Moreover, the existing research centers on provincial regions rather than municipal regions (as research conducted at the level of prefecture-level cities is more specific and closer to actual circumstances). The literature mostly focuses on the regulation of the relationship between technological innovation and atmospheric environmental pollution, and attaches less attention to the regulation of green-technology innovation and atmospheric environmental pollution with the environment as a mediator. Given the inadequacies in the existing research, this paper investigates the problems of green-technology innovation and atmospheric environmental pollution, constructs the Panel Threshold Model and Spatial Durbin Model that represents the relationship between green-technology innovation and atmospheric environmental pollution, and clarifies the atmospheric pollution reduction effects of green-technology innovation. Additionally, based on regional differences in the selection of data samples, this paper first explores the distribution characteristics of the samples of green-technology innovation, and proceeds to deeply analyze the heterogeneous impact or effect of green-technology innovation on atmospheric environmental pollution in different regions.

## 2. Research Hypotheses and Design

### 2.1. Research Hypotheses

Many factors need to be considered in the research on whether green-technology innovation reduces atmospheric environmental pollution, including environmental regulation, economic development, industrial transfer and agglomeration, and governmental regulation. The difference in the level of regional economic development also affects the introduction of green-technology innovation, after which the spillover effect of green-technology innovation extends to neighboring regions and forms an external factor. Therefore, this paper focuses on the relationship between green-technology innovation and atmospheric environmental pollution, the spatial spillover effect, and threshold characteristic, which possesses practical significance.

**Hypothesis 1:**  *The innovation of green technology is advantageous in mitigating atmospheric pollution.*

In economic development, atmospheric environmental pollution presents the biggest problem, and green-technology innovation forms an important measure to reduce atmospheric environmental pollution. In technological development, enterprises often sacrifice the ecological environment in the pursuit of economic benefits, which not only impairs tectological balance, but also undermines environmental bearing capacity. Under such a background, green-technology innovation hedges against the impact on environmental bearing capacity. According to Ullah et al. (2021), the effects of technological innovation on environmental quality are asymmetric, with the positive impacts exceeding the negative ones. Specifically, by enhancing energy efficiency, reducing waste, and promoting sustainable development, technological innovation can significantly reduce environmental pollution [21]. The influencing factors of environmental pollution basically comprise scale effect, structure effect, and technology effect. Among them, the technological effect plays the most important role in improving environmental quality. More advanced technology means less pollution (Grossman and Krueger, 1991) [22]. The effect of production technology manifests itself in the facts that technological progress will improve the production efficiency and resource utilization of enterprises, and that the saving of resources will reduce the damage to the environment, thus weakening environmental pollution. The effect of treatment technology lies in the fact that the improvement of production equipment and pollution-treatment equipment can reduce the emission of pollutants. Naturally, less emission of pollutants helps to reduce the emission of exhaust gas in the air, which alleviates atmospheric environmental pollution. The effect of structural optimization testifies to the fact that technological progress promotes the optimization and upgrading of industrial structure, eliminates the secondary industry, and actively develops the modern service industry and high-tech industry. This boosts the upgrading of industrial structure, reshapes the production model of industry, and definitely improves environmental quality. Simultaneously, green-technology innovation lowers the cost of environmental protection and fosters the growth of the green economy (David Popp, 2012) [23]. Gradually, the problem of environmental pollution becomes one of the most prominent problems and arouses the attention of various parties of green-technology innovation. Therefore, they tend to use scientific and technological means to solve the problem of environmental pollution. In the development of technological innovation, the integration of the idea of sustainable and ecology-oriented scientific and technological innovation helps to mitigate environmental pollution (Liu Guomai et al., 2020) [24].

Grounded in the above analysis, this paper proposes research Hypothesis 1: green-technology innovation can reduce atmospheric environmental pollution.

**Hypothesis 2:**  *The spatial spillover effect of green-technology innovation on atmospheric environmental pollution.*

Green-technology innovation functions as an effective means to realize the construction of ecological civilization. For some regions, the improvement of green-technology innovation reduces atmospheric environmental pollution in both these regions and neighboring regions, forming inter-regional interactions and spatial spillover effects (a spatial spillover is an essential form of spatial interaction, which manifests itself in the diffusion of socioeconomic factors in space). The reason lies in the fact that local industries play a learner role in the development and operation, and continuously learn and practice advanced approaches in green-technology innovation in neighboring regions. The knowledge in technological innovation has viscosity, both spatially and geographically (knowledge stickiness refers to the adhesive effect on knowledge caused by various factors, including the nature of knowledge (causal ambiguity and unverifiability), the source of knowledge (lack of incentives and unreliability), the recipient of knowledge (lack of absorption and retention abilities), and the knowledge transfer environment (lack of organizational environment and fragile internal connections)), with geographical distance proportional to the cost of knowledge dissemination (Wu Yuming, 2006) [25], thus enhancing regional capacity in green-technology innovation. Moreover, when the level of green-technology innovation in neighboring regions improves, the resource-utilization rate will increase, and the pollutant-emission rate will decrease accordingly. To put it another way, the reduction of atmospheric environmental pollution in neighboring regions indirectly eases local atmospheric environmental pollution (Lu Xuehuan et al., 2020) [10]. Evidently, as the level of green-technology innovation in neighboring regions improves, governments take more effective measures to control atmospheric environmental pollution, tighten the intensity of environmental regulation, and release corresponding policies on the control of atmospheric environmental pollution. If relevant policies in neighboring regions are well-implemented, local governments will naturally learn the policies on environmental regulation from neighboring regions, which has an indirect impact on the local treatment of atmospheric environmental pollution. For instance, in 2015, the Chinese National Development and Reform Commission issued a policy on the construction of demonstration cities and counties for a circular economy (http://www.gov.cn/xinwen/2015-09/24/content_2938128.htm (accessed on 10 March 2023)). Noteworthily, strengthening green-technology innovation in neighboring regions probably raises higher requirements for local policies, which urges local governments to participate in the strategic competition of environmental-protection policies, having a negative impact on local treatment of atmospheric environmental pollution (Li Zihao, 2016) [26].

Grounded in the above analysis, this paper proposes research Hypothesis 2: the rise of the level of local green-technology innovation helps to reduce atmospheric environmental pollution in neighboring regions, with a significant spatial spillover effect.

**Hypothesis 3:**  *The threshold characteristic of green-technology innovation on atmospheric environmental pollution.*

As governments and citizens pay more attention to environmental protection, the role of environmental regulation becomes increasingly prominent, which promotes the development of green-technology innovation, enables the market to meet the requirements of environmental regulation, and reduces environmental pollution (Li Fengqi, 2021) [27]. The innovation compensation effect of environmental regulation propels enterprises to carry out green-technology innovation, and the intensification of environmental regulation increases the expenditure of enterprises. In a way, this enhances the capacity of green-technology innovation (Lin Chunyan et al., 2019) [28]. When the intensity of environmental regulation remains low, the marginal cost of corporate environmental pollution proves less than the marginal profit, with the lack of driving force for the progress of green technology. When the intensity of environmental regulation rises, environmental regulation occasions the phenomenon of “the survival of the fittest” among enterprises (Jin Pei, 2009) [29]. To offset more production costs caused by greater intensity of environmental regulation, some enterprises take the initiative in green-technology innovation, thus strengthening the capacity of green-technology innovation (Jiang Fuxin et al., 2013) [30]. This enables enterprises to launch clean production and reduce atmospheric environmental pollution. Simultaneously, market-incentivized environmental regulation, which hinges on such means as pollution-fee income and environmental tax, increases the costs of enterprises with economic incentives and compels enterprises to promote pollution treatment and technological innovation. The model embodies “Polluter Pays Principle” and indirectly achieves the purpose of improving environmental quality (Fan Dan et al., 2020) [31]. Environmental regulation has a significant effect on reducing the atmospheric environmental pollution. Admittedly, under the impact of economic growth, environmental regulation probably produces the reverse effect. Yet, the impact of environmental regulation on the reduction of the emission of atmospheric environmental pollution gradually changes. With economic development, citizens raise their living standards and enhance their awareness of environmental protection, from which the impact of environmental regulation arises. Certainly, the reinforcement of the intensity of environmental regulation does not mean the automatic improvement of atmospheric environmental pollution. What matters is to establish and improve the overall system of environmental regulation.

Grounded in the above analysis, this paper proposes research Hypothesis 3: when environmental regulation reaches a higher level, the effect of green-technology innovation on reducing atmospheric environmental pollution proves more significant.

### 2.2. Research Design

#### 2.2.1. Model Construction

(1) Model Construction I:

Xiang Kun et al. (2015) constructed a Non-Spatial Interaction Model and a Spatial Durbin Model to test the economic drivers of fog-haze pollution based on six-year interval data (1985, 1990, 1995, 2000, 2005, 2010). As evidenced, the increase in coal consumption will worsen fog-haze pollution in one region and its neighboring regions, while the increase in power consumption can alleviate local fog-haze pollution and aggravate fog-haze pollution in neighboring regions [32]. Shao Shuai et al. (2016) conducted empirical identification and discussed corresponding haze-control policies on key factors that affect fog-haze pollution via the Dynamic Spatial Panel Model and System Generalized Method of Moments. As the results suggest, there is a significant spatial spillover effect of fog-haze pollution in provinces in China [33]. Based on the panel data of 285 prefecture-level and above cities in China, Cheng Zhonghua et al. (2019) constructed a Dynamic Spatial Panel Model to empirically analyze the impact of industrial-structure adjustment and technological progress on the reduction of fog-haze emission [34]. Based on the research of existing literature, this paper uses the Spatial Panel Regression Model to analyze the spatial spillover effect of green-technology innovation on atmospheric environmental pollution.

1. The Spatial Autoregression Model is expressed as follows:

(1) $${lnsmog1}_{it}=\rho \sum_{j=1}^{N}W_{it}\;{lnsmog1}_{it}+\beta_{1}{lner1}_{it}+\beta_{2}{lnden}_{it}+\beta_{3}{lnuba}_{it}+\beta_{4}{lney}_{it}+\beta_{5}{lnin}_{it}+\mu_{i}+\lambda_{i}+\varepsilon_{it}$$

In particular, ρ stands for the spatial lag (autoregression) coefficient. $W_{it}$ stands for the i-row and j-column elements of the normalized non-negative spatial weight matrix W in the N ∗ N dimension. The subscripts *i* and *t* stand for the year *t* of the *i*th province, respectively. $\mu_{i}$ and $\lambda_{i}$ stand for space (individual) effect and time effect, respectively.

2. The Spatial Durbin Model is expressed as follows:

(2) $$\begin{array}{cc} {lnsmog}_{it}= & \rho \sum_{j=1}^{N}W_{it}\;{lnsmog}_{it}+\beta_{1}{lnden}_{it}+\beta_{2}{lnuba}_{it}+\beta_{3}{lney}_{it}+\beta_{4}{lnin}_{it}\\ & \;\;\;\;+\beta_{5}\sum_{j=1}^{N}W_{it}\;{lnden}_{it}+\beta_{6}\sum_{j=1}^{N}W_{it}\;{lnuba}_{it}+\beta_{7}\sum_{j=1}^{N}W_{it}\;{lney}_{it}\\ & \;\;\;\;+\beta_{8}\sum_{j=1}^{N}\;W_{it}{lnin}_{it}+\mu_{i}+\lambda_{i}+\varepsilon_{it} \end{array}$$

In particular, ρ stands for the spatial regression coefficient. $W_{it}$ stands for the i-row and j-column elements of the normalized non-negative spatial weight matrix W in the N ∗ N dimension. The subscripts *i* and *t* stand for the year *t* of the *i*th province, respectively. $\mu_{i}$ and $\lambda_{i}$ stand for space (individual) effect and time effect, respectively.

(2) Model Construction II:

Guan Aiping et al. (2014) constructed a Non-Dynamic Panel Threshold Regression Model in their research on the impact of industrial transfer on technological innovation, to empirically test the technological spillover effect of inter-regional industrial transfer on developing western regions, as well as the impact of the absorptive capacity index on the technological spillover effect from the angles of economic development level, human capital, and technological gap of the transfer-in regions. As concluded, the inter-regional industrial transfer has a positive technological spillover effect on developing western regions [35]. Qin Bingtao et al. (2019) used the Threshold Panel Regression Model to analyze the relationship between the transfer of high-pollution industries and environmental pollution by taking the relative intensity of environmental regulation as the threshold variable. As the results show, there is a progressive-increase nonlinear relationship between the transfer of high-pollution industries and environmental pollution. As the intensity of relative environmental regulation jumps from a low threshold to a high threshold, the problem of environmental pollution caused by the transfer of high-pollution industries intensifies [36]. By combing the existing literature, this paper uses Hansen’s Threshold Regression Model to analyze the data and determine the threshold value. This paper explores the relationship between green-technology innovation and atmospheric environmental pollution, and takes environmental regulation as the threshold value to clarify the impact of green-technology innovation on atmospheric environmental pollution. The threshold model is expressed as follows:

(3) $$\begin{array}{cc} & {lnsmog}_{it}=\beta_{0}+\beta_{1}er\left({lner}_{it}<\gamma \right)+\beta_{2}{lner}_{it}\left({lner}_{it}\geq \gamma \right)+\beta_{3}{lngt}_{it}+\beta_{4}{lnden}_{it}+\beta_{5}{lnuba}_{it}+\beta_{6}{lney}_{it}+\beta_{7}{lnin}_{it}\\ & \;\;\;\;\;\;\;\;\;\;\;\;\;\;\;\;\;\;\;\;\;\;\;\;\;\;+\varepsilon_{it} \end{array}$$

In particular, β stands for the constant term. lnsmog stands for the natural logarithm of atmospheric environmental pollution. ln er stands for environmental regulation. $\varepsilon_{it}$ stands for the disturbance term. As delineated in Table 1, Control variables include population density, built-up area, economic development level, and industrial structure, respectively.

#### 2.2.2. Variable Description

(1) Explanatory Variable

This paper chooses green-technology innovation as the explanatory variable, expressed by the number of green-patent authorizations. The World Intellectual Property Organization classified green patents in the IPC Green Inventory released in 2010 (Wang Zhenyu et al., 2020) [37]. The China National Intellectual Property Administration defines the idea of the green patent as “patents for inventions, utility models and registered designs under the theme of green technologies conducive to resource conservation, energy-efficiency improvement and pollution prevention and control”. Green technology plays an important part in ecological civilization and serves as a major factor to coordinate ecology and economy and lead green development (Wang Banban et al., 2019) [38]. This paper chooses green-patent authorization rather than green-patent application, because the former better represents the innovation achieved in one region.

(2) Explained Variable

Following the construction method of Zhu Pingfang et al. (2011) [39] on atmospheric environmental pollution comprehensive index, this paper uses the comprehensive index (industrial sulfur dioxide/GDP + industrial smoke or powder dust/GDP + PM2.5/GDP)/3 to represent atmospheric environmental pollution and takes atmospheric environmental pollution as the explained variable.

(3) Threshold Variable

This paper takes environmental regulation as the threshold variable. Atmospheric environmental pollution mostly comes from the emission of gaseous pollutants in the production of industrial enterprises, so governmental control of gaseous pollutants can effectively measure regional environmental regulation. Following the practice of Shen Kunrong et al. (2017), this paper expresses environmental regulation with the removal amount of industrial sulfur dioxide in various regions [40].

(4) Control Variable
  1. Population density. The increase in population density probably promotes energy consumption and causes more pollution. The increase in population basically tallies with the growing trend of the emissions of sulfur dioxide and carbon. Notably, the impact of population density on environmental pollution depends on the dominant role of the scale effect and agglomeration effect (Liang Rui et al., 2020) [41].
  2. Built-up area. The built-up area refers to the area within an urban administrative area that has actually been developed and constructed in a large area, with municipal public facilities and public facilities basically available. The expansion of urban built-up areas has an impact on urban traffic, and the traffic demand increases with the expansion of the radius of the built-up area, thus emitting massive automobile exhaust and damaging the atmospheric environment (Hu Xuan, 2015) [42].
  3. Economic development level. This paper uses per capita GDP to measure the level of regional economic development. The level of regional economic development determines the consumption of natural resources to a large extent, and economic growth improves people’s living standards and consumption levels, which enhances their awareness of environmental protection and advances the reduction of environmental pollution.
  4. Industrial structure. This paper measures the proportion of the added value of the secondary industry in the local GDP in various regions or cities. The industrialization process spawns a significant increase in fossil-energy consumption and construction dust. The expansion of secondary-industry capacity undoubtedly constitutes an important source of fog-haze pollution (Lu Xuehuan et al., 2020) [10].

#### 2.2.3. Data Source and Variable Description & Statistics

Given the availability of data, this paper chooses 286 prefecture-level cities in China from 2005 to 2018 as the research object. Specifically, the data on fog-haze pollution of regions or cities come from the data of the annual mean of global PM2.5 concentration based on satellite monitoring published by the Socioeconomic Data and Applications Center of Columbia University(https://beta.sedac.ciesin.columbia.edu/, accessed on 10 March 2023). The emissions of industrial sulfur dioxide and industrial smoke (powder) dust as well as the removal of industrial sulfur dioxide in regions or cities come from the China Urban Statistical Yearbook and provincial statistical yearbooks, which are manually sorted by the author of this paper. The amount of green-patent authorization comes from the China National Intellectual Property Administration, which is manually sorted by the author of this paper. The data on indicators such as population density, built-up area, level of economic development, and industrial structure are sourced from the China Statistical Yearbook. Also Variable Description & Statistics is explicated in Table 2.

## 3. Empirical Analysis

### 3.1. The Empirical Analysis of Spatial Spillover Effect

Based on the data of 30 provinces, autonomous regions, and municipalities directly under the Central Government in China from 2005 to 2018, this paper constructs a Spatial Autoregression Model and Spatial Durbin Model to analyze the relationship between green-technology innovation and atmospheric environmental pollution, drawing the conclusion that green-technology innovation forms a significant spatial correlation with atmospheric environmental pollution. In other words, the level of atmospheric environmental pollution in one region is affected by the level (or emission) of atmospheric environmental pollution in neighboring regions.

The results of the Spatial Autoregression Model roughly accord with that of the Spatial Durbin Model; therefore, this paper mainly analyzes the results of the Spatial Durbin Model. As shown in Table 3, Table 4, Table 5 and Table 6, the main regression coefficient of the Spatial Durbin Model determines whether the direct effect proves significant, positive, or negative, so this paper combines the main regression coefficient with the cause of the direct effect. The main regression coefficient and direct effect of green-technology innovation both prove significantly negative, which demonstrates that green-technology innovation can effectively address the emission of environmental pollution. The spatial spillover of green-technology innovation proves significantly negative. One possible reason is that the development of local green-technology innovation significantly improves the production efficiency of enterprises and attracts enterprises from neighboring regions to agglomerate in the region, which reduces the emission of environmental pollutants in neighboring regions. Additionally, green-technology innovation can fundamentally reduce the emission of pollutants, upgrade the equipment and technology of industrial production, and then help to reduce the emission of pollutants in neighboring regions. The main regression coefficient and the direct effect of population density prove positive yet insignificant, which demonstrates that the pressure from the increase of local population density on atmospheric environmental pollution in one region and its neighboring regions proves insignificant, with no direct relationship. That is because the increase in population density slightly increases regional energy consumption and actualizes governmental treatment policies, with an insignificant impact on atmospheric environmental pollution. The main regression coefficient and the direct effect of the built-up area prove significantly negative, because the increase of local built-up area facilitates the transfer of pollution industries from neighboring regions, reduces the number of pollution industries in neighboring regions, and improves atmospheric environmental pollution in these regions. Additionally, owing to the expansion of the built-up area, the local energy-conservation industry multiplies, and the emission of pollutants from the energy-conservation industry decreases, which mitigates local atmospheric environmental pollution. The main regression coefficient and the direct effect of economic development level prove significantly negative, which demonstrates that China starts to change the production model of economic growth at the expense of the environment. The rise of the economic development level spurs enterprises to replace pollution-generation equipment with pollution-reduction equipment. A higher level of economic development means better control of pollution emissions and more reduction of environmental pollution. The main regression coefficient and the direct effect of industrial structure prove significantly positive, which demonstrates that the growth of the secondary industry aggravates atmospheric environmental pollution in neighboring regions. The flow of human and capital resources among various industries has a significant spatial correlation, and atmospheric environmental pollution per se has a spatial correlation, so the expansion of the secondary industry augments the pressure on one region and its neighboring regions in the treatment of atmospheric environmental pollution.

The regression coefficient of the indirect effect of green-technology innovation on atmospheric environmental pollution proves negative yet insignificant. This demonstrates that the introduction of green-technology innovation helps to alleviate atmospheric environmental pollution in neighboring regions, with unsatisfactory effect. With less attention to green-technology innovation, many enterprises uphold the logic of “Treatment after Pollution” and pursue short-term profits. As a result, atmospheric environmental pollution becomes more serious, and green-technology innovation alone can hardly slow down the pollution. Simultaneously, green-technology progress requires high costs. Local enterprises hope to “hitch a ride” and heavily rely on enterprises in neighboring regions. Therefore, green-technology progress in neighboring regions has a weak effect on the mitigation of atmospheric environmental pollution in one region. The indirect effect of the built-up area on atmospheric environmental pollution proves significantly negative, which demonstrates that the increase of the built-up area can reduce atmospheric environmental pollution in neighboring regions. After the expansion of the built-up area, motors and other vehicles that emit exhaust gas scatter and no longer produce massive exhaust gas, which reduces atmospheric environmental pollution in neighboring regions. Moreover, the expansion of built-up areas usually involves adding a large amount of non-building areas or green spaces, so the expansion of built-up areas will also increase the coverage of green areas, and neighboring regions learn to optimize the construction of green areas, which accelerates the treatment of environmental pollution in neighboring regions. The indirect effect of economic development level on atmospheric environmental pollution proves significantly negative, which demonstrates that the rise of economic development level makes people’s lives better. With their demands in daily life satisfied, people start to focus on their health. Since atmospheric environmental pollution means a major threat to their health, they hope relevant measures shall be taken to reduce it. Meanwhile, the rise of the economic development level materializes the increase of investment in the environmental treatment and the reinforcement of governmental policies on environmental regulation. Under such circumstances, neighboring regions will gain advanced experience, which helps to reduce atmospheric environmental pollution in neighboring regions. The indirect effect of industrial structure on atmospheric environmental pollution proves significantly negative because the change of industrial structure (with spatial effect) entails changes of talents, technologies, and resources. The spillover effect of talents, technologies, and resources urges enterprises in neighboring regions to learn and accept relevant development models, which will reduce the capital cost of industrial-structure adjustment in neighboring regions and increase the corresponding capital cost of pollution treatment. Likewise, the improvement of environmental quality depends on the upgrading and optimization of urban industrial structures. The reasonable transformation of industrial structures can effectively improve the atmospheric environment and mitigate environmental pollution in neighboring regions.

The empirical analysis of the Spatial Autoregression Model and Spatial Durbin Model verifies Hypothesis 1 (i.e., green-technology innovation can reduce atmospheric environmental pollution) and Hypothesis 2 (i.e., the rise of local green-technology innovation helps to reduce atmospheric environmental pollution in neighboring regions and produces a significant spatial spillover effect).

### 3.2. Threshold Characteristic

The single threshold value of environmental regulation reaches 11.6373, and the double threshold value of environmental regulation reaches 11.7913 and 10.2717. When the F value of the double threshold reaches 28.02 and 24.5, it proves significant at the level of 5%, with a double threshold effect.

As shown in Table 7, environmental regulation has the characteristic of double threshold. When environmental regulation reaches lower than 10.2717, the regression coefficient of environmental regulation on atmospheric environmental pollution proves positive yet insignificant. This demonstrates that environmental regulation has a weak impact on the relationship between green-technology innovation and atmospheric environmental pollution at the initial stage of implementation. One reason is that at the early stage, policies on environmental regulation are neither well-designed nor thoroughly implemented. Coupled with other factors, the intensity of environmental regulation remains low and has an insignificant regulatory effect on atmospheric environmental pollution. The level of green-technology innovation also improves slightly. When environmental regulation reaches higher than 10.2717 yet lower than 11.7913, the regression coefficient of environmental regulation on atmospheric environmental pollution proves negative and insignificant. This is because with the improvement of environmental regulation, China attaches more importance to green-technology innovation and atmospheric environmental pollution and invests financial funds for the upgrading of green-technology equipment, the change of pollution-emission means, and the improvement of production technology. Under such a background, enterprises emit less pollution and reduce atmospheric environmental pollution. At the transitional stage, however, green-technology innovation has a limited inhibitory effect on atmospheric environmental pollution. When environmental regulation reaches higher than 11.7913, the regression coefficient of environmental regulation on atmospheric environmental pollution proves significant and reaches −0.078. This demonstrates that with the rise of environmental regulation, green-technology innovation has a greater inhibitory effect on atmospheric environmental pollution. The fact that China pays attention to environmental regulation quickens the maturity of green-technology innovation. The inhibitory effect of green-technology innovation on environmental pollution mainly exists in the effect of production technology, the effect of treatment technology, and the effect of structural optimization. Specifically, the effect of production technology stems from the progress of production technology, which improves the utilization rate of resources and energy in industrial production, tremendously saves or effectively utilizes resources, and reduces pollutant emissions accordingly. The effect of treatment technology comes from the progress of green technology, including the use of clean energy and pollution-treatment equipment in production as well as a green and environment-friendly production process, which decreases the emission of pollutants. The effect of structural optimization refers to the fact that the progress of green technology promotes the structural optimization and upgrading of industrial enterprises and achieves the reduction of pollutant emissions in terms of overall industrial scale and structure. Besides, green-technology innovation advances, with a significant impact on the reduction of pollutant emissions. Therefore, enterprises should invest more capital or attach more attention to green technology, choose large-scale areas for the development of green-technology innovation, and make better use of green technology to produce a learning effect, so that green technology extends to more fields and plays a better role in curbing pollutant emissions.

Therefore, the above analysis verifies Hypothesis 3: when environmental regulation reaches a higher level, the effect of green-technology innovation on reducing atmospheric environmental pollution becomes more significant.

## 4. Robustness Test: A Case Study of the Yangtze River Delta

Following the practice of Liu Chenyue et al. (2017) [43], this paper replaces the explained variable of atmospheric environmental pollution and uses annual fog-haze pollution (pm) in various cities to measure the atmospheric environmental pollution and conduct a robustness test.

The single threshold value of environmental regulation reaches 11.0739, and the double threshold value of environmental regulation reaches 11.4563 and 10.6199.

As shown in Table 8, after replacing the explained variable, green-technology innovation in one region and neighboring regions still significantly improves the treatment of atmospheric environmental pollution, and the spatial spillover effect generally agrees with the above research hypotheses. Simultaneously, green-technology innovation has a significant double threshold effect on fog-haze pollution in terms of environmental regulation. Environmental regulation plays an effective role in curbing fog-haze pollution vis green-technology innovation. Therefore, the major results in this paper prove to be robust and reliable.

## 5. Heterogeneity Analysis

### Case Studies of the Beijing-Tianjin-Hebei Region, the Yangtze River Delta, the Pearl River Delta, the Chengdu-Chongqing Region and Northeastern Region

This paper completes the heterogeneity test of the Beijing-Tianjin-Hebei Region, the Yangtze River Delta, the Pearl River Delta, the Chengdu-Chongqing Region, and the Northeastern Region with the Spatial Measurement Model.

As shown in Table 9, in the Yangtze River Delta, the Pearl River Delta and Beijing-Tianjin-Hebei Region, the main regression coefficient and the direct effect of green-technology innovation prove significantly negative; yet, in the Pearl River Delta and Beijing-Tianjin-Hebei Region, the indirect effect of green-technology innovation proves insignificant. In the Northeastern Region, the main regression coefficient and the direct effect of green-technology innovation prove positive and insignificant, and the indirect effect of green-technology innovation proves negative and insignificant. In the Chengdu-Chongqing Region, the main regression coefficient of green-technology innovation proves negative and insignificant, and the direct effect and indirect effect of green-technology innovation prove negative and significant.

In the Yangtze River Delta, the Pearl River Delta, and the Beijing-Tianjin-Hebei Region, the main regression coefficient and the direct effect of green-technology innovation prove significantly negative. This is mainly because the Yangtze River Delta, which covers Shanghai, Jiangsu, Zhejiang, and Anhui, boasts advanced economic development, with a high level of economic openness. The development level of a green economy in the Yangtze River Delta is higher than that in the Beijing-Tianjin-Hebei Region and Northeastern Region. Shanghai and Zhejiang have taken the lead in green-technology innovation in China. The industrial structure of Shanghai is dominated by the tertiary industry, which emits fewer industrial pollutants. Compared with Jiangsu, Zhejiang, and Anhui, Shanghai invests the most capital for the treatment of environmental pollution. Therefore, Shanghai plays a decisive role in promoting the level of green-technology innovation in the Yangtze River Delta. Noticeably, in 2018, to accelerate the construction of a complete local legal system for environmental protection, Shanghai revised local laws and regulations such as The Regulations for the Prevention & Control of Atmospheric Pollution in Shanghai, which mitigates atmospheric environmental pollution to a certain extent. Zhejiang rapidly improves the level of Internet information, and Hangzhou becomes a major base for new media culture in China. Zhejiang and Jiangsu vigorously develop the secondary industry and introduce many industrial products; however, the spatial spillover effect of green-technology innovation can effectively reduce atmospheric environmental pollution. Meanwhile, in the Yangtze River Delta, residents embody a high educational level, and the investment to scientific research proves sufficient, which raises the regional technological level and promotes the development of green-technology innovation. Green-technology innovation in the Yangtze River Delta produces a spatial spillover effect, which effectively drives the development of relatively backward cities in the region, improves the level of green-technology innovation in the region and its neighboring regions, and reduces the impact on atmospheric environmental pollution. Vis-à-vis the Yangtze River Delta, the Pearl River Delta sees faster economic development and enjoys better air quality than the Yangtze River Delta and Beijing-Tianjin-Hebei Region. The development of high-tech enterprises in Shenzhen, Guangzhou, Dongguan, and other cities significantly heightens regional green productivity, and achieves remarkable results in the transformation and upgrading of the manufacturing industry. Scientific and technological innovation serves as a major driving force for high-quality economic growth. Moreover, there are many coastal cities in the Pearl River Delta, where air quality is better than the interior region. The amount of wind and rainfall is also an important factor that reduces local atmospheric environmental pollution. However, there are numerous high-tech enterprises and high-end manufacturing industries as well as traditional manufacturing industries in the Pearl River Delta, which restricts the spillover effect of green-technology innovation. Developing cities may not actively learn from developed cities in green-technology innovation, and green-technology innovation plays an insignificant role in reducing the atmospheric environmental pollution in neighboring regions. Covering Beijing, Tianjin, and Hebei, the Beijing-Tianjin-Hebei Region suffers the most serious atmospheric environmental pollution in China. The Beijing-Tianjin-Hebei Region mainly adopts the development model of an extensive industry that features high energy consumption, high pollution, and high input. Compared with the Yangtze River Delta and the Pearl River Delta, the Beijing-Tianjin-Hebei Region has fewer high-tech industries, especially in Hebei. As a circum-capital urban cluster, the industrial structure and economic development of Beijing, Tianjin, and Hebei are subject to governmental policies. In the 21st century, the development of Beijing and Tianjin relies on support from neighboring cities. With the best geographical location, Hebei undertakes the industrial transfer from Beijing and Tianjin. Furthermore, Hebei possesses abundant mineral resources and supports the development of Beijing and Tianjin, which exacerbates atmospheric environmental pollution in Hebei. Simultaneously, extensive industries near Beijing are successively shut down, and the emission of pollutants decreases accordingly. This happens later in Tianjin and Hebei, so atmospheric environmental pollution in Hebei remains serious. Located in the Circum-Bohai Sea Economic Zone, the Beijing-Tianjin-Hebei Region has high-level openness and introduces more green technologies. Enterprises in Beijing, Tianjin, and Hebei exchange ideas on green technology, which makes green technology mature and alleviates local atmospheric environmental pollution. Yet, neighboring regions of the Beijing-Tianjin-Hebei Region attach less attention to green-technology innovation. The Yangtze River Delta and the Pearl River Delta see rapid economic development and great progress in green-technology innovation. In contrast, in the Beijing-Tianjin-Hebei Region, green-technology innovation remains at a low level, which limits the learning effect in neighboring regions. In other words, green-technology innovation plays a minor role in attenuating atmospheric environmental pollution in neighboring regions. As a result, the coefficient of green-technology innovation in the Beijing-Tianjin-Hebei Region proves insignificant.

Different from the Yangtze River Delta, the Pearl River Delta, and Beijing-Tianjin-Hebei Region, the Northeastern Region and Chengdu-Chongqing Region see an insignificant spatial effect. Firstly, in the Northeastern Region, the main regression coefficient and the direct effect prove positive and insignificant, and the indirect effect proves negative and insignificant. This is because in economic development, the Northeastern Region lags far behind the Yangtze River Delta, the Pearl River Delta, the Beijing-Tianjin-Hebei Region, and the Chengdu-Chongqing Region. The Northeastern Region includes Heilongjiang, Jilin, and Liaoning provinces as well as three cities and one league of Inner Mongolia. With a low level of green-technology innovation, the Northeastern Region is beset with a high proportion of brain drain, high-level talents, and technological backbone in particular. Owing to the unsatisfactory policy environment in the Northeastern Region, the adequacy rates of talent and technology remain low. The Northeastern Region functions as the “granary” of China. In agriculture, it focuses on traditional farming and stockbreeding that make low demands on green-technology innovation. In the Northeastern Region, the economy develops slowly, green-technology innovation remains at a low level, and many high-level talents leave. Simultaneously, there is a great quantity of industrial enterprises. Therefore, in the Northeastern Region, green-technology innovation plays a minor role in reducing the emission of pollutants from industrial enterprises and mitigating atmospheric environmental pollution. The main regression coefficient and the direct effect prove positive and insignificant. In spite of this, the learning effect of green-technology innovation allows neighboring regions to introduce green-technology innovation, which reaches a low level yet has an inhibitory impact on neighboring regions. In contrast, in the Chengdu-Chongqing Region, the main regression coefficient of green-technology innovation proves negative yet insignificant, and the direct effect and indirect effect of green-technology innovation prove significantly negative. This is because the Chengdu-Chongqing Region forges the most developed economy in western China and forms a major urban belt in China. In terms of the overall level of green-technology innovation, the Chengdu-Chongqing Region rivals the Beijing-Tianjin-Hebei Region. However, the level of scientific and technological innovation in various cities in the Chengdu-Chongqing Region is significantly polarized. In Chengdu and Chongqing, there are more scientific research institutes, high-tech enterprises, and R and D investment to scientific and technological innovation than in other cities in the region, with a significantly higher level of scientific and technological innovation. The low level of scientific and technological innovation in other cities has an adverse effect on the overall level in the region. Most cities in the Chengdu-Chongqing Region stand in the Sichuan Basin, with unique geographical conditions. Atmospheric pollution arises from complex causes or seasonal factors, which makes it hard to improve the atmospheric environment. Moreover, in the Chengdu-Chongqing Region, atmospheric environmental pollution embodies high spatial correlation. Chongqing enjoys better air quality. Therefore, in the region, the main regression coefficients of green-technology innovation and atmospheric environmental pollution prove insignificant. Conspicuously, the level of green-technology innovation constantly increases, and the funds for green-technology innovation continuously enlarge. With more attention given to green-technology innovation, the scale effect of green-technology innovation alleviates atmospheric environmental pollution in the Chengdu-Chongqing Region. Simultaneously, neighboring regions continue to learn from green-technology innovation in the Chengdu-Chongqing Region, which weakens atmospheric environment pollution in the neighboring regions.

As the above analysis suggests, there are obvious differences in the level of green-technology innovation, the investment to R and D, the level of economic development, geographical location, and air quality among the Yangtze River Delta, the Pearl River Delta, the Beijing-Tianjin-Hebei Region, the Northeastern Region, and the Chengdu-Chongqing Region. Therefore, the degree of atmospheric environmental pollution in these regions varies, with significant heterogenicity in the impact of green-technology innovation on atmospheric environmental pollution.

## 6. Conclusions and Policy-Related Suggestions

### 6.1. Research Conclusions

Based on the panel data of 30 provinces, autonomous regions, and municipalities directly under the Central Government in China from 2005 to 2018, this paper constructs a Spatial Autoregression Model and Spatial Durbin Model to analyze the relationship between green-technology innovation and atmospheric environmental pollution. This paper concludes at three levels. Firstly, green-technology innovation has a significant inhibitory effect on atmospheric environmental pollution, which mainly exists in the effect of production technology, the effect of treatment technology, and the effect of structural optimization. Secondly, green-technology innovation has a significant spatial spillover effect on atmospheric environmental pollution. When local industries give play to the learning effect and continue to learn and practice advanced approaches in green-technology innovation in neighboring regions, local capability in green-technology innovation will remarkably rise. Thirdly, when the intensity of environmental regulation reaches its value, green-technology innovation can effectively inhibit atmospheric environmental pollution. When environmental regulation reaches lower than the threshold, the effect of environmental regulation on atmospheric environmental pollution proves insignificant.

### 6.2. Policy-Related Suggestions

#### 6.2.1. Fostering Green Technology Innovation for Environmental Sustainability and Economic Growth

Enhancing green technology innovation can help reduce atmospheric pollution. To strengthen green-technology innovation for the treatment of the atmospheric environment, local governments need to increase the investment to the research and development of green-technology innovation, expedite the research and development of green technology, promote the application of green-technology innovation in production, and reduce the use cost of green-technology innovation. Moreover, high-quality talents play an indispensable role in the development of green-technology innovation. Local governments can formulate favorable policies on talent-introduction and settlement, consider how to attract and introduce high-quality talents, and enhance the green acuity of high-quality talents, so as to disseminate the idea of green development among the public. As clear property rights mobilize enterprises to participate in green-technology innovation, local governments should highlight the protection of green intellectual property systems, establish strict supervision mechanisms, and protect the achievements of green-technology innovation. Additionally, local governments should quicken the research and development of environmental-protection technologies and institutions, integrate green technology into corporate development and people’s daily life, and accelerate the realization of green and coordinated development of the economy and environment.

#### 6.2.2. Leveraging High-Level Green-Tech Innovation Regions for Coordinated Development and Knowledge Exchange

Augmenting the supporting role of regions with a high level of green-technology innovation and fostering the coordinated development of neighboring regions are important goals. The improvement of the level of green-technology innovation necessitates the implementation of the development strategy of “one leads two”. To firstly strengthen the policy support for regions with high-level green-technology innovation and then drive the coordinated development of neighboring regions enlarges the coverage of green-technology innovation. Specifically, governments should strengthen the exchanges on the knowledge of green-technology innovation. Governments of regions with high-level green-technology innovation can reinforce the publicity of green-technology innovation to give play to the learning effect of green-technology innovation, so that neighboring regions learn the gist of green technology. Additionally, enterprises should deepen technological cooperation and exchanges in green-technology innovation. Enterprises in regions with high-level green-technology innovation can promote the research and development of clean technologies for energy conservation and emission reduction and pollution prevention, so as to gain profits and arouse the research and development interest of enterprises in neighboring regions. Governments should also establish a joint prevention and control mechanism for atmospheric environmental pollution, and work together with neighboring regions to design, formulate, and implement the plans for the treatment of atmospheric environmental pollution, develop the treatment system of green-technology innovation, and share the achievements of green-technology innovation, so as to produce the spillover effect of treatment policies and results.

#### 6.2.3. Enhancing Environmental Regulation to Encourage Green-Tech Innovation in Heavily Polluted Regions

We highlight the importance of boosting up the intensity of environmental regulation in heavily polluted regions and making full use of the inhibitory effect of green-technology innovation. Governments should promote the development of green-technology innovation with the policies on environmental regulation. In line with the degree of regional pollution, local governments can adopt different policies on environmental regulation and prevent atmospheric environmental pollution with appropriate methods, so as to formulate policies suitable for various regions and effectively avoid a one-size-fits-all approach. To give full play to the role of different environmental-regulation tools in green-technology innovation, governments should vigorously support market-oriented incentive policies, which play a significant role in boosting green-technology innovation and produce an external effect via environmental tax, emission trading licenses, and other means. In terms of mandatory regulatory tools such as command and control that are difficult to practice, governments can make reasonable planning to achieve ideal treatment. Lastly, regions with more serious environmental pollution must tighten the policies on environmental regulation. Local governments ought to focus on the intensity of the policies on environmental regulation in heavily polluted regions, actively design the policies on environmental regulation to promote the development of green-technology innovation, and provide relevant subsidies to enterprises in the use of green technology to encourage enterprises to expand the application of green technology.

## Figures and Tables

**Table 1:** Variable Description.

Name	Name Description
smog	Atmospheric Environmental Pollution
gt	Green-Technology Innovation
er	Environmental Regulation
den	Population Density
uba	Built-up Area
ey	Economic Development Level
in	Industrial Structure

**Table 2:** Variable Description and Statistics.

Variable	Sample Size	Mean Value	Standard Deviation	The Minimum Value	The Maximum Value
smog	3990	−21.08713	2.28609	−27.25011	0.00000
gt	3990	2.92953	4.67108	−23.02585	9.49830
er	3990	10.32545	2.18277	−11.51293	16.72783
den	3990	7.94237	0.80561	3.29584	9.92010
uba	3990	4.36596	0.87574	1.87947	7.97903
ey	3990	10.31334	0.77566	4.59512	15.67520
in	3990	3.84681	0.24964	2.19723	4.51053

**Table 3:** Main regression coefficients for spatial effects (Main).

	Lny	
Main		Wx
Lnx	−0.013 ***	0.001
	(−2.622)	(0.439)
lnm1	0.007	−0.007
	(0.199)	(−0.586)
lnm2	−0.540 ***	−0.096 ***
	(−6.907)	(−2.943)
lnm3	−1.401 ***	0.096 ***
	(−19.672)	(4.873)
lnm4	0.737 ***	−0.207 ***
	(4.587)	(−4.398)
Spatial		
rho	0.098 ***	
	(31.649)	
Variance		
sigma2_e	1.061 ***	
	(43.870)	
N	3990	
R2	0.030	

t statistics in parentheses. *** *p* < 0.01.

**Table 4:** Spatial autocorrelation direct effects (LR_Direct).

LR_Direct	Lnpy
Lnx	−0.013 **
	(−2.304)
lnm1	0.001
	(0.029)
lnm2	−0.636 ***
	(−7.808)
lnm3	−1.433 ***
	(−20.773)
lnm4	0.647 ***
	(4.248)

t statistics in parentheses. ** *p* < 0.05, *** *p* < 0.01.

**Table 5:** The indirect spatial autocorrelation effects (LR_Indirect).

LR_Indirect	Lnpy
Lnx	−0.001
	(−0.062)
lnm1	−0.071
	(−0.528)
lnm2	−1.615 ***
	(−4.654)
lnm3	−0.442 **
	(−2.509)
lnm4	−1.436 ***
	(−3.164)

t statistics in parentheses. ** *p* < 0.05, *** *p* < 0.01.

**Table 6:** The total spatial autocorrelation effect (LR_Total).

LR_Total	Lnpy
Lnx	−0.014
	(−0.526)
lnm1	−0.070
	(−0.468)
lnm2	−2.250 ***
	(−5.787)
lnm3	−1.875 ***
	(−9.438)
lnm4	−0.789 *
	(−1.651)
N	3990
R2	0.030

t statistics in parentheses. * *p* < 0.1, *** *p* < 0.01.

**Table 7:** The Regression Results of Threshold Characteristic.

	**Lnsmog**	**Lnsmog**
lngt	−0.018 ***	−0.018 ***
	(−3.149)	(−3.144)
lnden	−0.055	−0.064
	(−1.329)	(−1.556)
lnuba	−0.762 ***	−0.755 ***
	(−8.120)	(−8.062)
lney	−2.177 ***	−2.127 ***
	(−37.271)	(−35.913)
lnin	0.691 ***	0.686 ***
	(4.170)	(4.144)
0._cat#c.lner	−0.044 **	0.005
	(−2.386)	(0.244)
1._cat#c.lner	−0.078 ***	−0.028
	(−4.370)	(−1.492)
2._cat#c.lner		−0.061 ***
		(−3.359)
_cons	3.096 ***	2.348 ***
	(3.865)	(2.876)
N	3990	3990
R2	0.581	0.583
adj. R2	0.548	0.550

t statistics in parentheses. ** *p* < 0.05, *** *p* < 0.01.

**Table 8:** Robustness Test.

	The Threshold Characteristic of Panel Threshold Model		Spatial Durbin Model		
			Total Effect	Direct Effect	Indirect Effect
	lnpm	lnpm	lnpm	lnpm	lnpm
lngt	−0.010 ***	−0.009 ***	−0.007 ***	−0.006 *	0.010
	(−3.244)	(−2.973)	(−2.726)	(−1.945)	(0.943)
lnden	0.035 ***	0.035 ***	0.016 *	0.015 *	−0.007
	(3.247)	(3.314)	(1.905)	(1.706)	(−0.210)
lnuba	−0.055 ***	−0.057 ***	−0.030 ***	−0.056 ***	−0.320 ***
	(−3.741)	(−3.941)	(−2.613)	(−4.280)	(−5.199)
lney	−0.079 ***	−0.087 ***	−0.040 ***	−0.039 ***	0.011
	(−6.201)	(−6.790)	(−2.854)	(−2.835)	(0.274)
lnin	0.421 ***	0.446 ***	0.213 ***	0.183 ***	−0.361 ***
	(10.718)	(11.335)	(5.673)	(4.653)	(−3.206)
0._cat#c.lner	−0.009 **	−0.017 ***			
	(−2.088)	(−3.577)			
1._cat#c.lner	−0.003	−0.011 ***			
	(−0.719)	(−2.607)			
2._cat#c.lner		−0.006			
		(−1.342)			
_cons	3.021 ***	3.069 ***	2.713 ***		
	(14.836)	(15.131)	(13.638)		
Spatial			0.141 ***	0.129 ***	
rho			(33.144)	(25.782)	
Spatial					
rho			0.009 ***	0.011 ***	
			(25.660)	(23.830)	
				−2.699 ***	
				(−24.212)	
N	1386	1386	1386	1386	
R2	0.297	0.309	0.000	0.001	
adj. R2	0.240	0.251			

t statistics in parentheses. * *p* < 0.1, ** *p* < 0.05, *** *p* < 0.01.

**Table 9:** The heterogeneity analysis of the Spatial Durbin Model.

	The Yangtze River Delta	The Pearl River Delta	Beijing-Tianjin-Hebei Region	Northeastern Region	Chengdu- Chongqing Region
	lnsmog	lnsmog	lnsmog	lnsmog	lnsmog
Total Effect					
lngt	−0.068 ***	−0.568 ***	−0.772 ***	0.012	−0.008
	(−3.861)	(−2.831)	(−7.309)	(0.498)	(−0.697)
lnden	−0.101	0.052	0.190	0.076	−0.095
	(−1.061)	(0.276)	(1.306)	(0.732)	(−1.080)
lnuba	−0.076	0.361	0.823 ***	0.054	−1.539 ***
	(−0.969)	(1.126)	(5.418)	(0.251)	(−7.443)
lney	−1.309 ***	−1.029 ***	−1.100 ***	−2.959 ***	−0.134
	(−9.805)	(−3.309)	(−4.309)	(−11.116)	(−1.560)
lnin	3.329 ***	2.456 ***	0.768	2.909 ***	−1.767 ***
	(11.988)	(3.979)	(1.614)	(6.792)	(−4.726)
_cons	−19.079 ***	−19.766 ***	−14.956 ***	−1.901	−4.829 ***
	(−12.436)	(−5.416)	(−4.543)	(−0.846)	(−3.001)
N	574	126	182	490	266
R2	0.676	0.744	0.764	0.441	0.382
Direct Effect					
lngt	−0.138 ***	−0.638 ***	−0.820 ***	0.012	−0.045 ***
	(−5.924)	(−3.201)	(−5.889)	(0.459)	(−2.643)
lnden	0.005	0.051	0.170	0.076	−0.080
	(0.043)	(0.245)	(0.857)	(0.767)	(−0.924)
lnuba	0.054	0.394	1.002 ***	0.126	−1.559 ***
	(0.549)	(0.966)	(5.013)	(0.570)	(−6.459)
lney	−1.252 ***	−0.949 ***	−1.048 ***	−2.979 ***	−0.117
	(−9.204)	(−3.049)	(−3.475)	(−12.015)	(−1.087)
lnin	3.613 ***	2.951 ***	1.129 *	2.881 ***	−1.575 ***
	(12.081)	(3.699)	(1.659)	(7.053)	(−3.955)
Indirect Effect					
lngt	−0.959 ***	−0.586	−0.489	−0.026	−0.404 ***
	(−6.143)	(−1.523)	(−0.839)	(−0.445)	(−4.182)
lnden	1.490 **	0.048	−0.123	0.092	0.210
	(2.383)	(0.092)	(−0.157)	(0.455)	(0.725)
lnuba	1.659 ***	0.078	1.551	1.048	−0.450
	(2.628)	(0.049)	(1.605)	(1.390)	(−0.438)
lney	0.856 *	0.715	0.665	−0.230	0.227
	(1.728)	(1.207)	(0.549)	(−0.601)	(0.420)
lnin	3.766 ***	3.506	3.079	−0.564	2.165
	(2.924)	(1.565)	(1.021)	(−0.927)	(1.476)

t statistics in parentheses. * *p* < 0.1, ** *p* < 0.05, *** *p* < 0.01.

## Data Availability

The data on fog-haze pollution of regions or cities come from the data of the annual mean of global PM2.5 concentration based on satellite monitoring published by the Socioeconomic Data and Applications Center of Columbia University (https://beta.sedac.ciesin.columbia.edu/ assessed on 10 March 2023). The emissions of industrial sulfur dioxide and industrial smoke (powder) dust as well as the removal of industrial sulfur dioxide in regions or cities come from the China Urban Statistical Yearbook and provincial statistical yearbooks, which are manually sorted by the author of this paper. The amount of green-patent authorization comes from the China National Intellectual Property Administration, which is manually sorted by the author of this paper. The data on indicators such as population density, built-up area, level of economic development, and industrial structure are sourced from the China Statistical Yearbook.
